# Temperature Field in the Heat Transfer Process of PEEK Thermoplastic Composite Fiber Placement

**DOI:** 10.3390/ma13194417

**Published:** 2020-10-04

**Authors:** Zhongliang Cao, Mingjun Dong, Kailei Liu, Hongya Fu

**Affiliations:** 1 School of Mechanical Engineering, Jiangsu University of Technology, Changzhou 213001, China; monjohns@outlook.com (M.D.); lkl@jsut.edu.cn (K.L.); 2 School of Mechatronics Engineering, Harbin Institute of Technology, Harbin 150001, China; hongyafu@hit.edu.cn

**Keywords:** thermoplastic fiber, temperature field, PEEK, automatic placement

## Abstract

Under the effect of different process parameters, the temperature field inside the thermoplastic fiber is very complex and directly affects the fusion quality between the resins. Considering the heat transfer behavior of thermoplastic fiber polyether ether ketone (PEEK) as the research object, a mathematical model of heat transfer in the thermoplastic composite fiber placement with the relevant boundary conditions was established. Ansys Parametric Design Language (APDL) was used to generate the finite element model and simulate the transient process, not only to explore the influence of various process parameters on the temperature field, but also to build an online temperature field measurement system. The influence rules of placement process parameters and mold initial temperature with respect to the temperature field in the first layer were obtained. Combining the relationship between heating temperature and placement speed, when the first layer was laid, the placement process temperature could be quickly reached by low speed and high temperature. The temperature data were collected by the online detection system. Compared with the temperature data from the simulation, the error was below 8%, which verified the correctness of the heat transfer model. The academic research results will lay a theoretical foundation for the thermoplastic fiber placement.

## 1. Introduction

Since fiber-reinforced composite materials have the advantages of light weight, high strength, high modulus, non-reflection of electromagnetic waves, and large design freedom, they have become the primary materials for aerospace components and large aircrafts [1,2,3]. The materials used for automatic fiber placement are mainly resin-based fiber composite materials, which can be divided into thermosetting composite materials and thermoplastic composite materials according to the different resin matrix materials. The former are widely used as high-strength and lightweight materials in various fields such as aerospace, ships, and transportation [4,5,6,7]. However, during the molding process, the “autoclave curing” technology is limited by the size of the processing site and components, and it suffers from high energy and time consumption. These shortcomings limit the application of thermosetting composite materials [8,9,10]. Compared with thermosetting composite materials, the requirements for curing thermoplastic composite materials are lower. They also have the advantages of good weldability, impact toughness, cyclability, and chemical resistance, making thermoplastic composites the research focus in the field of composite molding [11,12,13,14].

**Figure 1:**
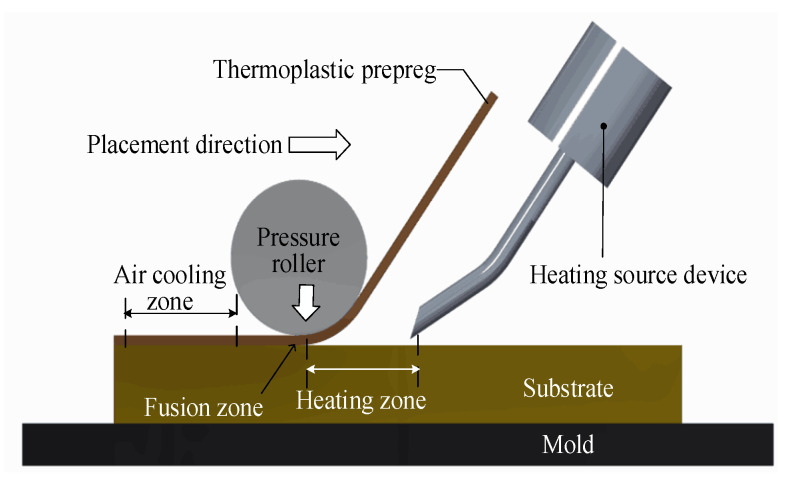
Schematic diagram of automatic thermoplastic fiber placement.

Thermoplastic fiber placement uses in situ curing technology, applying appropriate heat sources and pressure to the thermoplastic fibers to manufacture the component. The in situ curing technology adds curing heat sources during the preforming process. Thus, the heat source, which directly affects the placement effect and efficiency, is the key to the thermoplastic fiber placement process [15]. When the thermoplastic resin matrix reaches its melting point, the molecular chains move, which causes the resin matrix to change physically and to have a certain viscoelasticity. Due to the influence of external pressure, fusion of the thermoplastic fiber between the current layer and existing layers occurs [16,17]. The combination of thermoplastic composite materials and in situ curing technology significantly reduces the production cost and improves the production efficiency and the quality of composite material components. The temperature in the layer directly affects the fusion quality of the layer. When the thermoplastic resin matrix is exposed to high temperature, the change in its internal temperature is a nonlinear process. Boeing and Airbus have made research plans on thermoplastic composite materials for structural parts of aircraft, aiming to expand their application for the main load-bearing parts [18,19,20]. The automatic placement process of thermoplastic fibers is shown in Figure 1. The thermoplastic fiber placement process is mainly divided into the heating zone, fusion zone, and natural air-cooling zone. The purpose of heating is to make the first layer of thermoplastic fiber and the substrate layer reach a molten state together, becoming completely fused under the pressure roller. During this process, the molecular chains penetrate each other, diffuse into each other, and finally form an integrated part. Then, the substrate layer enters the natural air-cooling zone to undergo cooling and consolidation.

In the process of thermoplastic fiber placement, the heat transfer model is the primary model to start with as part of the process modeling with the resulting temperature [21,22,23,24]. At present, hot-air heating is widely used in the field of automatic placement of composite materials due to its low cost and small heating window. Research scholars established heat transfer models on the basis of relevant factors such as the type of heating source, the inherent characteristics of the fiber material, and the temperature of the placement environment, thereby finally solving the model. John et al. [25] used high-temperature nitrogen for thermoplastic fiber placement and studied the effects of placement speed, heating temperature, heating time, placement pressure, and heating head height on the fusion strength between the layers of the composite board. The heat transfer speed is faster in the thickness direction than in the width direction. Ghasemi et al. [26] established a two-dimensional heat transfer model for automatic fiber placement and discussed the placement speed, heat source temperature, heat source width, and other process parameters, ultimately obtaining the influence law of the placement process parameters with respect to the temperature field. The results showed that it is beneficial to improve the fusion strength when the substrate layer is heated to a temperature above the melting point multiple times, but this also increases the porosity between the layers. Lionetto et al. [27] established a simple two-dimensional model of thermoplastic prepreg tape, studied the temperature field change process, and conducted experimental measurements. The experimental results were consistent with the simulation. In recent years, other heating methods, such as ultrasound, infrared, laser, and electron beam, also passed test experiments and are widely used. Laser heating has become the main heating source due to its advantages of large energy density, high efficiency, and fast response time. Ultrasonic heating converts the high-frequency electrical energy into mechanical energy through the transducer conversion device, allowing the fibers to rub against each other to generate heat. Infrared heating is based on the principle of electromagnetic radiation heat transfer, with the disadvantage of long heating time. Dipa et al. [28] analyzed the strength properties of laminates obtained via the lay-up method and laser-assisted heating, aiming to determine the influence of laser-assisted heating on the crystallinity and the strength of laminates. Stokes et al. [29,30] conducted experimental analysis using laser heating, and obtained the temperature field change process of the first prepreg tape and the substrate layer through a long-wave infrared camera. Li et al. [31] established a heat transfer model. The related process parameters of the placement of thermoplastic fibers, the cooling crystallinity during the deposition of thermoplastic fibers, and the effects of cooling temperature and cooling time on the crystallinity were analyzed and discussed. Furthermore, the effect of preheating on the placement speed was studied, but relevant demonstration experiments were not carried out.

There are three main types of heat transfer models: one-dimensional, two-dimensional, and three-dimensional heat transfer models [17,25,26,27,28,32,33,34,35,36]. Because the three-dimensional heat transfer model is relatively complex and computationally expensive, few studies considering the three-dimensional behavior of the process have been performed, whereas the first two models are more widely used. From the literature review, different heat transfer models were established on the basis of different conditions and analyzed under specific circumstances, whereby many assumptions were introduced to simplify the problem. The main purpose was to obtain the temperature field distribution of the laminate and then analyze the crystallinity of the final product and the performance index of the laminate. The initial temperature of the mold during the thermoplastic fiber placement process has a great influence on the heat transfer during the first-layer placement process, during which the heat transfer speed is very fast. Existing heat transfer models often ignore this impact on heat transfer, resulting in a large gap between analysis and reality. In addition, few researchers analyzed the temperature field changes in the first-layer placement. Most simply analyzed the temperature field in the existing substrate. In this paper, heat transfer during the placement of thermoplastic fibers (polyether ether ketone (AS4/PEEK), melting temperature 343 °C) was taken as the research object. A transient two-dimensional heat transfer model was established, and the relevant boundary conditions of the heat transfer model were determined. A finite element method (FEM) model of the temperature field was established using the Ansys Parametric Design Language (APDL). Then, the temperature gradient changes between the layers under the influence of the initial temperature of the mold, the temperature field changes of the substrate layer during the laying process, the relationship between the heating temperature and the temperature field of the layer, and the reasonable heating temperature were analyzed. A thermoplastic fiber placement platform was built to detect the temperature field in the layer and verify the accuracy of the model.

## 2. Heat Transfer Model of Thermoplastic Fiber Placement

### 2.1. Establishment of Thermal Conductivity Equation

In the thermoplastic fiber placement process, it is beneficial to construct a reasonable processing window if the temperature field change at any point can be correctly predicted. Because a single layer of thermoplastic prepreg is very thin (thickness of approximately 0.125 mm), the temperature after heating is much higher than its melting point temperature. At the same time, the temperature of the thermoplastic prepreg is higher than that of the laid layer. For these reasons, the analysis was focused on the temperature field in the substrate layer.

The heat transfer differential equation of anisotropic thermoplastic fibers per unit volume is shown in Equation (1).

(1) $$K_{x}\frac{\partial^{2}T}{\partial x^{2}}+K_{y}\frac{\partial^{2}T}{\partial y^{2}}+K_{z}\frac{\partial^{2}T}{\partial z^{2}}=\rho c\frac{\partial T}{\partial t},$$

where *K_x_* is the thermal conductivity of the thermoplastic fiber along the *x*-direction (W/(m·°C)), *K_y_* is the thermal conductivity of the thermoplastic fiber along the y direction (W/(m·°C)), *K_z_* is the thermal conductivity of the thermoplastic fibers along the z direction (W/(m·°C)), *ρ* is the density of the thermoplastic fiber (kg/m^3^), *c* is the specific heat capacity of the thermoplastic fiber (J/(kg·°C)), and *T* is the function of the temperature field, with regard to space coordinates and time.

**Figure 2:**
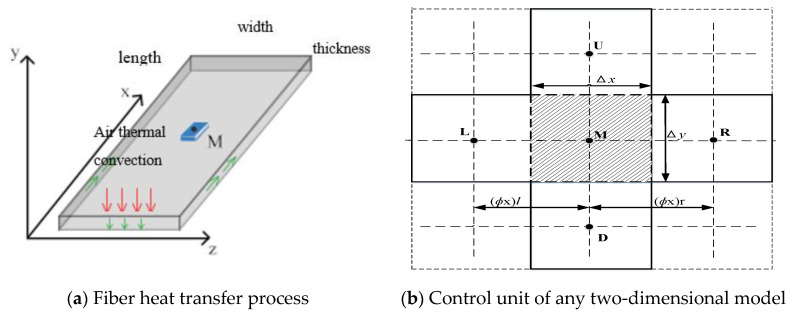
Schematic diagram of heat exchange of thermoplastic fibers.

The thermoplastic prepreg itself has a three-dimensional structure, and the heat transfers to all three dimensions during heating. Since the width of the heating source is greater than that of the thermoplastic prepreg in reality, the heat transfer of the thermoplastic prepreg can usually be considered uniform within its width. Therefore, the heat transfer in the width direction of the prepreg is ignored in the simulation analysis, and the heat is only transferred in the length and thickness directions during the placement process, as shown in Figure 2a, where M is the thermoplastic unit in the fiber body. High-temperature compressed hot air was selected as the heating source to generate forced air thermal convection on the surface of the thermoplastic prepreg. Convection heat exchange occurred in the body of the thermoplastic prepreg and natural heat transfer directly occurred between the lower surface of the thermoplastic prepreg and the adjacent layer. Therefore, heat was transferred to the underlying layer, resulting in a temperature gradient change. At the same time, heat diffused along the direction of the prepreg. In the subsequent finite element simulation, the calculation volume of the two-dimensional heat transfer model was smaller, and the calculation speed was faster. According to the heat transfer law, Equation (1) was transformed into a two-dimensional heat transfer equation [33,37].

(2) $$k_{x}\frac{\partial^{2}T}{\partial x^{2}}+k_{y}\frac{\partial^{2}T}{\partial y^{2}}=\rho c\frac{\partial T}{\partial t}$$

Figure 2b shows the control unit of any two-dimensional model in the thermoplastic fiber body. The heat is transferred from one unit to the adjacent unit in the upper, lower, left, and right directions. Point *M* in the figure represents the middle unit body. As indicated by the dotted line, point *L* represents the left unit of the middle unit body, point *R* represents the right unit of the middle unit body, point *U* represents the upper unit of the middle unit body, and point *D* represents the lower unit of the middle unit body. Equation (2) can, thus, be transformed into a discrete equation [38].

(3) $$a_{M}T_{M}=a_{R}T_{R}+a_{L}T_{L}+a_{U}T_{U}+a_{D}T_{D}+b$$

where:

$$\begin{array}{l} a_{R}=\frac{k_{r}\Delta y}{{\left(\partial x\right)}_{r}};a_{L}=\frac{k_{l}\Delta y}{{\left(\partial x\right)}_{l}};a_{U}=\frac{k_{u}\Delta x}{{\left(\partial y\right)}_{u}}\\ a_{D}=\frac{k_{d}\Delta x}{{\left(\partial y\right)}_{d}};b=a_{M}^{0}T_{M}^{0};a_{M}^{0}=\frac{\rho c\Delta x\Delta y}{\Delta t} \end{array}$$

where *k_r_*, *k_l_* is the thermal conductivity along the *x*-direction (W/(m·°C)), *k_u_*, *k_d_* is the thermal conductivity along the *y*-direction (W/(m·°C)), *T*^0^*_M_* is the initial temperature of the control unit (°C), *a*^0^*_M_* is the initial heat capacity of the control unit (J/(kg·°C)), and Δ*x*, Δ*y* is the size of the control unit along the *x-* and *y*-axes.

The thermoplastic fiber filament was dispersed into a limited number of control unit bodies, and the discrete control units exchanged heat with each other to complete the heat transfer. Macroscopically, the heat transfer was carried out along the length and thickness of the thermoplastic fiber filaments and, microscopically, heat was exchanged between the top, bottom, left, and right sides of the unit, thereby realizing heat conduction. Equation (3) was applied to the secondary development of the finite element model. The analysis of the placement temperature field was a transient analysis, i.e., solving the temperature field under the action of time-varying loads and boundary conditions. The initial temperature and boundary conditions were determined before the solution, whereby the boundary conditions were natural convection boundary conditions and the initial temperature was used as the initial condition of the first load step. During the calculation, the results of the previous load step were used as the initial conditions of the subsequent load step.

### 2.2. Heating Geometry Model and Boundary Conditions

In the thermoplastic fiber placement process, the temperature of the heating source is one of the most important process parameters, directly affecting the change in temperature field in the substrate layer. The melting point temperature of PEEK resin is 343 °C. It was verified by experiments that the optimal temperature in the placement process is 370–400 °C. If the temperature exceeds this range, the viscosity of thermoplastic resin decreases. When the temperature exceeds 580 °C, the interlaminar fusion strength is seriously affected, and the substrate layer may even be destroyed. In the heating process of thermoplastic fiber, the heat transfer process includes forced convection and heat transfer. Forced convection first occurs between the high-pressure hot air and the surface of the thermoplastic fiber, and then heat transfer occurs between the layers. Therefore, it is very important to establish a heating geometry model with high accuracy for the analysis of the temperature field in thermoplastic fiber placement.

**Figure 3:**
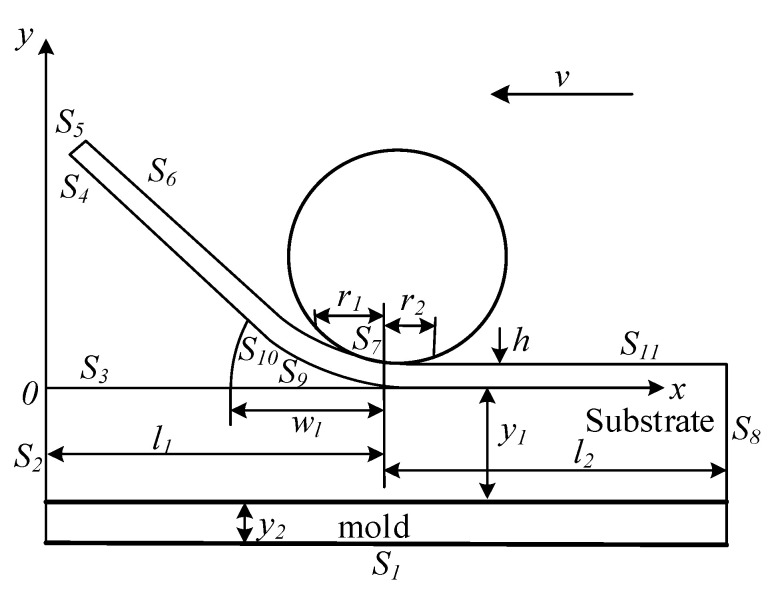
Geometric model of thermoplastic fiber automatic placement.

The change in temperature field in the thermoplastic fiber placement process is a transient process. The geometric model of thermoplastic fiber automatic placement is illustrated in Figure 3, as well as the corresponding boundaries, volume relationships, and coordinate positions. The coordinate system is located in the whole substrate and does not change with the placement forming process. The *y*-axis represents the thickness direction of the substrate vertically, with the origin set to the left side of the workpiece; the *x*-axis is set on the upper surface of the substrate, and the horizontal direction to the right is its positive direction. *l*_1_ is the distance from the nip point to the *y*-axis, *l*_2_ is the length of the melt region to the right boundary of the substrate, *y*_1_ is the thickness of the substrate, *y*_2_ is the thickness of the mold, *r*_1_ is the contact length along the *x*-axis between the roller and the fiber (the prepreg and the substrate are deformed during the action of the pressing roller, which causes the back of the pressing roller to contact the prepreg), *r*_2_ is the length along the *x*-axis between the back roller and the prepreg, *r*_1_ plus *r*_2_ is the total contact length of the pressing roller between the roller and the prepreg, w_l_ is the heating length of the heating source, and *h* is the thickness of the prepreg. *S*_i_ (i = 1, 2, …, 11) represents the boundary and position of each surface of the mold, the substrate, and the prepreg.

According to the heat transfer theory, the boundary conditions of the heat transfer model were determined. Before placement, it was assumed that the initial temperature of mold was *T*_tool_ and that of thermoplastic prepreg fiber was *T*_tow_. When *t* = 0, the initial temperature on the bottom surface S_1_ of the mold and on the left and right surfaces S_2_ and S_8_ of the substrate could be formulated as

T(x, y) = *T*_tool_ (4)

while that on the bottom surface S_4_, S_5_, S_6_ of the prepreg could be formulated as

T(x, y) = *T*_tow_ (5)

According to the Fourier heat equation and cooling law, the boundary conditions of each surface in the model are described below. On the bottom surface S_1_ of the mold, the heated mold was used as a heating source. The upper surface of the mold was in direct contact with the lowest surface of the substrate, while the lower surface of the mold was in direct contact with the outside air. The natural convection boundary conditions were as follows:

(6) $$K_{n}\frac{\partial T}{\partial n}\left(0\leq x\leq l_{1}+l_{2},-\left(y_{1}+y_{2}\right)\right)=-h_{a}\left(T-T_{a}\right)$$

where *h_a_* is the air heat transfer coefficient (W/(m·°C)), *T_a_* is the ambient temperature (25 °C), and *K_n_* is the thermal conductivity along the normal direction (W/(m·°C)).

In the geometric model, on the left surface *S*_2_ of the substrate, because the temperature as the same as that of the mold, the temperature was constant. It only contacted the outside air directly. The natural convection boundary conditions were as follows:

(7) $$T\left(0,-\left(y_{1}+y_{2}\right)\leq y\leq 0\right)=T_{\mathrm{tool}}$$

In the geometric model, the lower surface *S*_4_ of the prepreg and the upper surface *S*_3_ of the substrate were all directly convective with the air.

(8) $$K_{n}\frac{\partial T}{\partial n}\left(0\leq x<l_{1}-w_{l},0\right)=-h_{a}\left(T-T_{a}\right)$$

On the end surface *S*_5_ of the prepreg in the geometric model, the initial temperature of the thermoplastic prepreg was constant, and it only contacted the outside air directly. The natural convection boundary conditions were as follows:

(9) $$T\left(0,0\leq y\leq h\right)=T_{\mathrm{tow}}$$

In the geometric model, on the upper surface *S*_6_ of the prepreg, the thermoplastic prepreg only conducted direct convective heat transfer with the air, and the natural convective boundary conditions were as follows:

(10) $$K_{n}\frac{\partial T}{\partial n}\left(0\leq x<l_{1}-r_{1},h\right)=-h_{a}\left(T-T_{a}\right)$$

In the geometric model, on the upper surface *S*_11_ of the substrate, only direct convective heat transfer with air was carried out. The natural convective boundary conditions were as follows:

(11) $$K_{n}\frac{\partial T}{\partial n}\left(l_{1}+r_{2}<x\leq l_{1}+l_{2},h\right)=-h_{a}\left(T-T_{a}\right)$$

On the lower surface *S*_7_ of the pressure roller in the geometric model, due to the heat exchange between the thermoplastic prepreg and the pressure roller, the convection boundary conditions in the contact area were as follows:

(12) $$K_{n}\frac{\partial T}{\partial n}\left(l_{1}-r_{1}\leq x\leq l_{1}+r_{2},h\right)=-K_{r}\left(T-T_{r}\right)$$

where *K_r_* is the conductivity of the mold (W/(m·°C)), and *T_r_* is the initial temperature of the mold (°C).

In the geometric model, on the right surface *S*_8_ of the substrate, because the temperature was the same as that of the mold, the temperature was constant. It only contacted the outside air directly, and the natural convection boundary conditions were as follows:

(13) $$T\left(0,-\left(y_{1}+y_{2}\right)\leq y\leq 0\right)=T_{\mathrm{tool}}$$

In the geometric model, the upper surface S_9_ of the substrate and the lower surface *S*_10_ of the prepreg were the main heated areas in the heating zone. When high-temperature hot air acted on these two contact surfaces, the boundary conditions of convection heat transfer were as follows:

(14) $$K_{n}\frac{\partial T}{\partial n}\left(l_{1}-w_{l}\leq x\leq l_{1},0\right)=-h_{hg}\left(T-T_{hg}\right)$$

where *T_hg_* is the hot gas temperature (°C), and *h_hg_* is the hot gas heat transfer coefficient (W/(m·°C)).

## 3. Finite Element Modeling and Simulation Analysis

### 3.1. Finite Element Modeling and Solution of Heat Transfer Model

**Figure 4:**
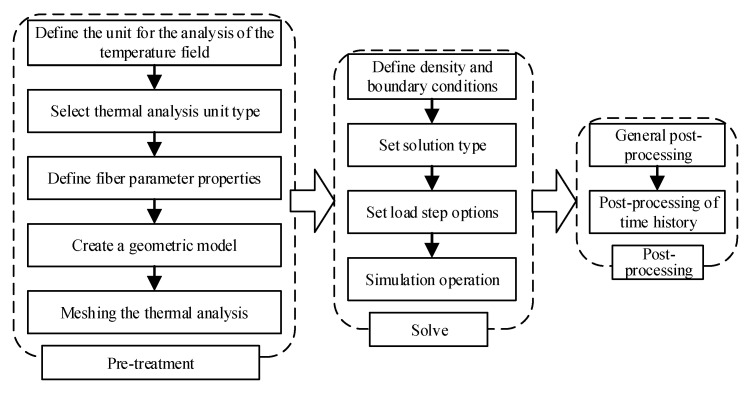
Temperature field analysis steps.

Thermoplastic fiber placement is a continuous process of gradual fusion between the prepreg and the substrate. Therefore, in the whole placement process, the temperature of the prepreg and the substrate at different positions changes continuously with time. In this paper, the finite element software Ansys was used to model and simulate the temperature field in the process of thermoplastic fiber placement. The specific steps of temperature field analysis during the placement process of thermoplastic fiber are shown in Figure 4.

Firstly, the unit of placement temperature field analysis was defined in the pre-processing module, and the type of thermal analysis unit was selected. A geometric model was created by setting the length of the model to 200 mm and the thickness of layers to 0.125 mm. The related material properties of the thermoplastic fiber and mold are defined in Table 1 and Table 2. The elements at equal intervals were meshed for thermal analysis. Secondly, the heat flux load, the boundary conditions of heat dissipation, and the load step options were set in the solution module. As the placement head moved during the placement, the size of the layer changed and the load calculation in the transient process was realized. Finally, the temperature change corresponding to each node in the finite element model was checked in the post-processing module.

Table 3 shows the relevant boundary conditions of the two-dimensional heat transfer model in the thermoplastic fiber placement process. The diameter and heating length of the pressure roller were determined by the design size of the placement equipment. The temperature of the pressure roller and the initial temperature of the mold were set to 80 °C and 150 °C, respectively, to increase the initial substrate temperature of the laminate, which was beneficial for the first layers of the thermoplastic fibers in the initial placement process to absorb more heat from the heat source and improve the fusion efficiency between the layers.

**Figure 5:**
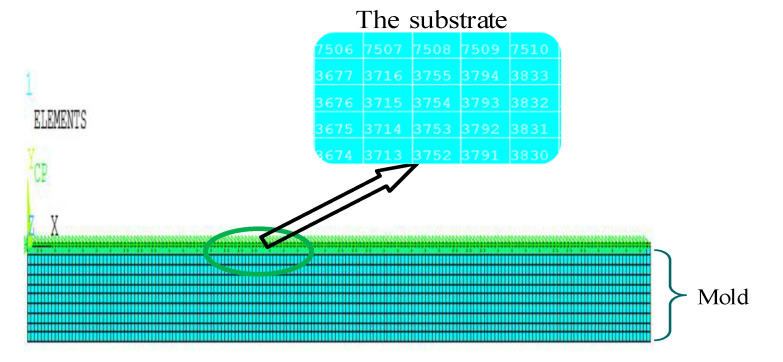
Finite element mesh model of layer and mold.

The meshing of the finite element model of the substrate and mold is illustrated in Figure 5. The thickness and length of the substrate were equally spaced, and two elements were meshed along the thickness direction of each layer. If the element size was smaller than that of the mold, the number of elements of the substrate was also greater and denser than that of the mold. Finally, the command flow was set by APDL, and the establishment of related models was completed.

### 3.2. Finite Element Simulation Results of Temperature Field

#### 3.2.1. Influence of Mold Temperature on the Temperature Field of the First Layer

In the actual placement process, because the mold had a certain volume and mass, heat exchange occurred during the heat transfer process and was approximately equivalent to a heat absorption source. During placement, a large amount of heat in the substrate was absorbed by the mold, which reduced the temperature in the resin, rendering it not conducive to interlaminar fusion. In existing research, the influence of the initial temperature of the mold on the temperature field is often ignored, and only the substrate is preheated to increase the placement temperature. When the first layer is laid, the mold as a cold source acts as a huge heat absorption source, and the temperature field of the first layer is greatly affected by the thermoplastic fiber during the placement process. Therefore, the initial temperature of the mold was studied in the temperature field of the first layer. This effect was very important, and its main purpose was to increase the temperature of the first layer and enhance the fusion quality.

**Figure 6:**
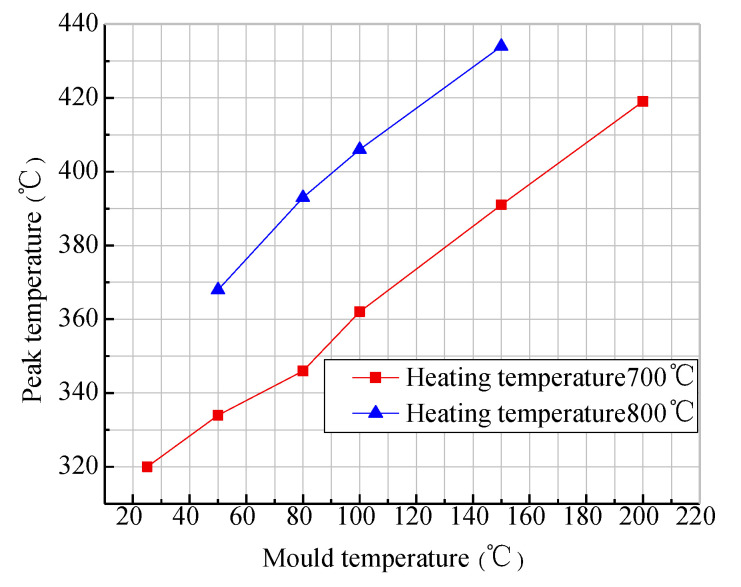
The change curve of peak temperature in the first layer under different mold temperatures.

The node in the middle of the layer was selected to extract its peak temperature. Figure 6 shows the change curve of the peak temperature of the first layer under different mold temperature conditions, where the placement speed was 10 mm/s, and the two heating temperature values were 700 °C and 800 °C. It can be seen from the figure that, when the heating temperature was 700 °C and the initial temperature of the mold reached 150 °C, the peak temperature of the first layer was only above 370 °C. If the heating temperature was increased to 800 °C, with an initial mold temperature of 80 °C, the peak temperature of the first layer could reach above 370 °C, and the thermoplastic prepreg could reach the temperature required by the placement process. It can also be seen from the analysis results that the initial temperature of the mold had a certain relationship with the heating temperature. When the heating temperature is too high, the single thermoplastic prepreg can cause pyrolysis (resin decomposition reaction) or even spontaneous combustion. Therefore, in the first-layer placement, the initial temperature of the mold should be appropriately increased and the heating temperature should be lowered to prevent the pyrolysis of the single thermoplastic prepreg, which is not conducive to the quality of the layer. Through the analysis of the peak temperature during the first-layer placement process, it was obtained that the initial temperature of the mold had a great influence on the temperature field. It was also mentioned in the literature [32,33] that the preheating temperature of the mold is ideally close to the glass transition temperature of the material at 143 °C, which is conducive to the crystallization of thermoplastic materials, whereas excessive high temperature is averse to the formation of the layers. The relationship between the initial temperature and the heating temperature of the mold is integrated. In this paper, simulation of the thermoplastic fiber placement process adopted heating the mold instead of preheating. For this process, the initial temperature of the mold was set to 150 °C without special instructions.

#### 3.2.2. The Influence of Heating Temperature on the Temperature Field of the First Layer

**Figure 7:**
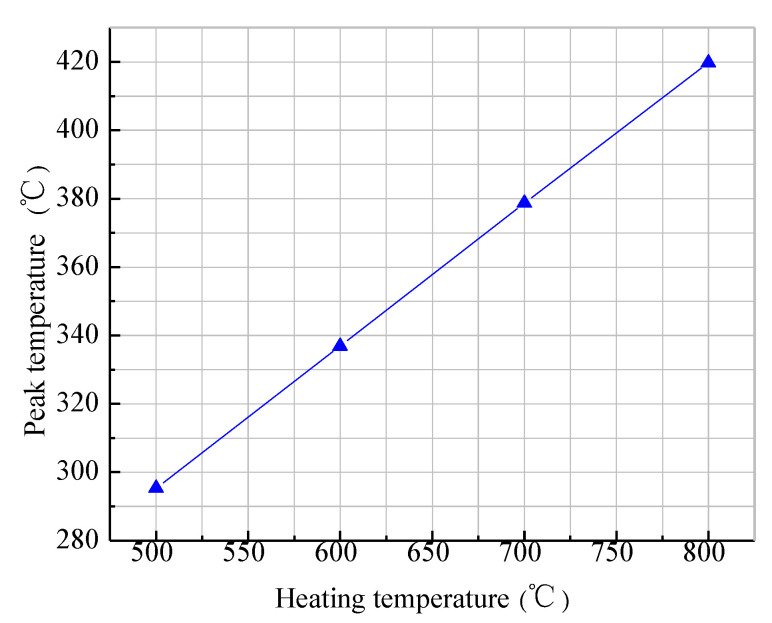
Variation curve of the peak temperature in the first layer under different heating temperatures.

After the initial temperature of the mold was set, the influence of the heating temperature on the peak temperature was analyzed in the first layer. The change curve of the peak temperature of the first layer under different heating temperature conditions is shown in Figure 7, with a placement speed of 10 mm/s. It can be seen from the figure that, when the heating temperature was below 600 °C, the temperature of the substrate layer could not reach the required placement process temperature (above 370 °C), and, when the heating temperature exceeded 700 °C, the thermoplastic prepreg fiber could reach the normal placement process temperature and achieve interlaminar fusion. This also shows that the preheating of the mold can improve the initial temperature in the first-layer placement, but the mold still absorbs an amount of heat in the layer. Without a high enough heating temperature, it was difficult for the temperature to attain the process temperature. Therefore, when in the first-layer placement, the heating temperature was increased (ideally above 750 °C), such that it was conducive to interlaminar fusion in the first-layer placement process. At the same time, considering the relationship between the heating temperature and the placement speed, for the first-layer placement, the temperature in the layers could quickly reach the process temperature under low placement speed and high heating temperature. This was beneficial for interlaminar fusion under the action of the pressing roller, laying a certain theoretical foundation for the subsequent placement experiments.

### 3.3. Influence of Heating Temperature on Temperature Field in the Substrate

**Figure 8:**
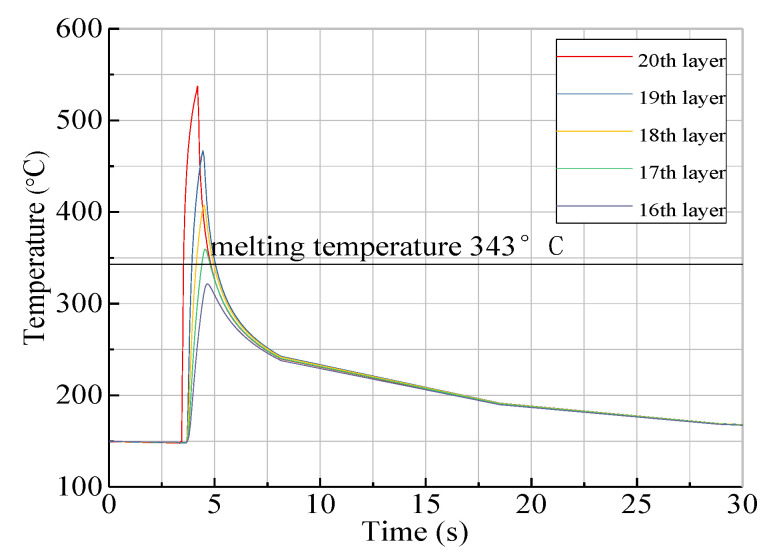
Temperature profiles of each layer at v = 20 mm/s and T = 700 °C.

**Figure 9:**
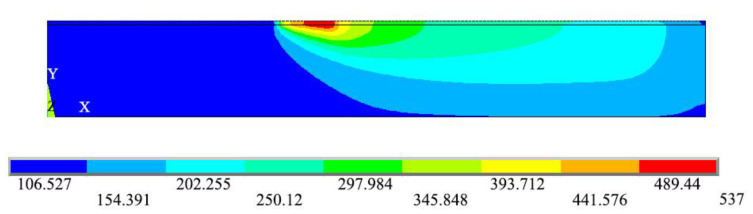
Temperature fields of each layer at v = 20 mm/s and T = 700 °C.

For thermoplastic fiber placement, the heating temperature is one of the key process parameters. The temperature gradient distribution of the substrate is mainly affected by the heating temperature. An appropriate heating temperature can improve the placement efficiency and obtain better fusion quality between layers. Temperature profiles of each layer are shown in Figure 8 with a placement speed of v = 20 mm/s and heating temperature of T = 700 °C, while the temperature fields of each layer are shown in Figure 9. The temperature curve of the 16th layer to 20th layer is illustrated in Figure 8, where the temperature change in any unit of the fiber body increased suddenly at the meshing point, reaching the maximum temperature value before gradually decreasing, with the peak temperature decreasing layer by layer. As seen in Figure 9, the temperature in the substrate obviously changed along the thickness and length directions, while it also showed a peak at the meshing point.

## 4. Experimental Verification and Analysis of Temperature Field

### 4.1. Experimenta Set-Up

**Figure 10:**
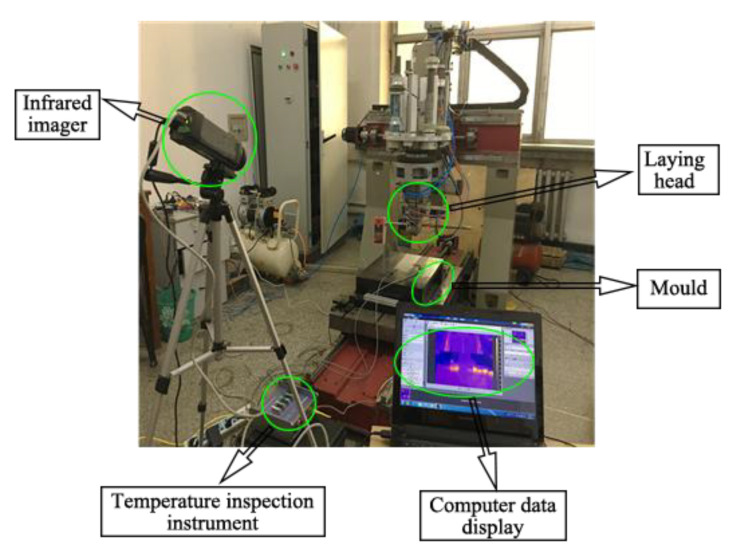
Physical diagram of the online temperature field measurement system.

In order to realize real-time measurements of temperature field changes in the substrate layer during the thermoplastic fiber placement process, an online temperature field measurement platform was established. This system consisted of three basic modules, namely, a temperature acquisition module, signal processing module, and data storage module. A physical diagram of the online temperature field measurement system is shown in Figure 10.

**Figure 11:**
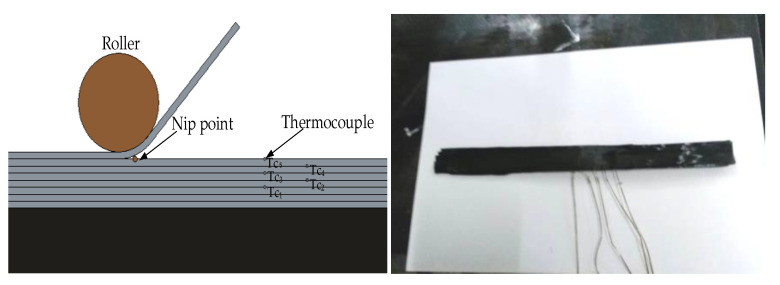
Type K thermocouple layout diagram and physical diagram.

It was essential to embed the thermocouple into the substrate to record internal temperature field variation during the placement process. Considering that the rate of temperature change within the thermoplastic fiber could reach values of 1000 °C/s, high-resolution thermocouples were chosen. In this paper, a fine metal wire type K thermocouple (nickel chromium, temperature range: 0–1200 °C, the measurement accuracy: ±2.5 °C) was embedded between each layer of laminate, which adopted an alternative arrangement to prevent an increase in the local thickness of laminates. Figure 11 shows the thermocouple layout. The temperature value of the substrate was processed by the temperature inspection instrument (model: STI-AS-DC-C40, measurement accuracy: 0.2%); then, the computer displayed its change process and stored relative data for subsequent processing.

**Figure 12:**
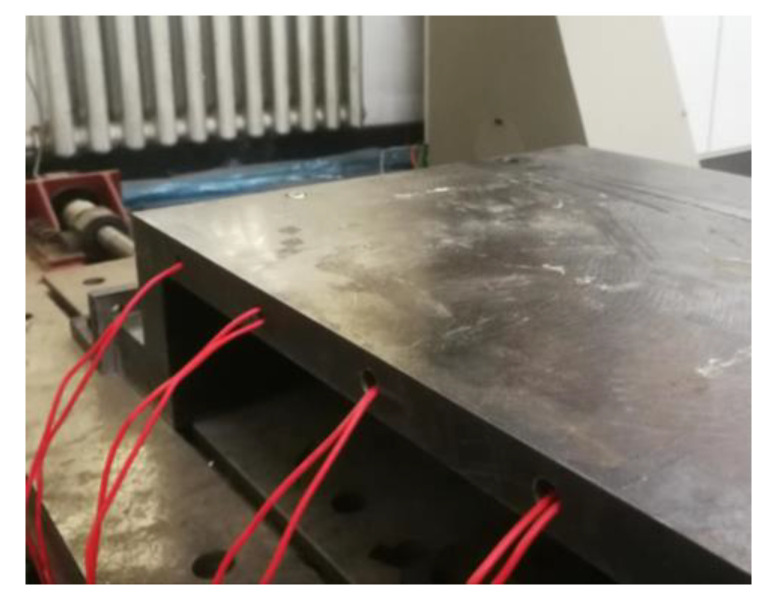
Mold heating diagram.

It could be derived from the simulation analysis that setting the initial temperature of the mold to 150 °C for the first-layer placement was beneficial for fusion quality improvement. On the one hand, the heat insulation effect on the substrate layer can be enhanced by an increase in layer number. On the other hand, the mold needs to keep the temperature of the bottom layer constant, which can always guarantee that the temperature of overall substrate layers is higher than the glass transition temperature. Considering the above reasons, a heating rod (pipe diameter: 10 mm) was utilized to heat the mold, maintaining the temperature at approximately 150 °C. Figure 12 shows a schematic diagram of mold heating. During the first-layer placement, the thermoplastic composite material could not be fused with the mold made of 45# steel. Therefore, a layer of high-temperature-resistant glue (resistance up to 1300 °C) was coated on the surface of the mold before placement, thereby ensuring that the first layer adhered to the mold, improving convenience for subsequent placement.

### 4.2. Experimental Result

**Figure 13:**
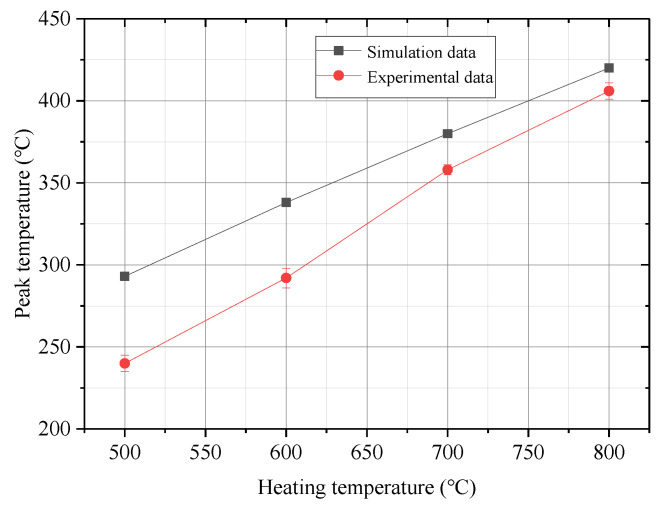
Comparison of the peak temperature of the first layer under different heating temperatures.

The number of experiments in each group was no less than five, and the average value was taken. The error range of the test results was ±6 °C. Figure 13 compares the peak temperature of the first layer under different heating temperatures with a placement speed of 10 mm/s and a mold temperature of 150 °C. It can be seen from the figure that the peak temperature increased as the heating temperature increased, but the measured peak temperature of the first layer in the experiment was lower than that obtained from simulation analysis. When the heating temperature reached 500 °C and 800 °C, the difference in peak temperature between the measured value and simulated value was approximately 54 °C and 16 °C, respectively.

**Figure 14:**
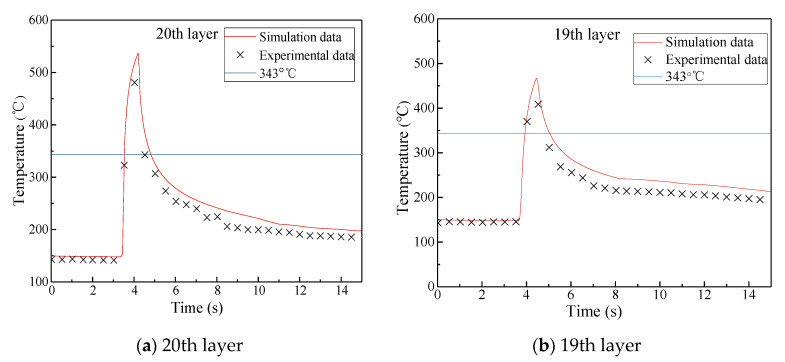

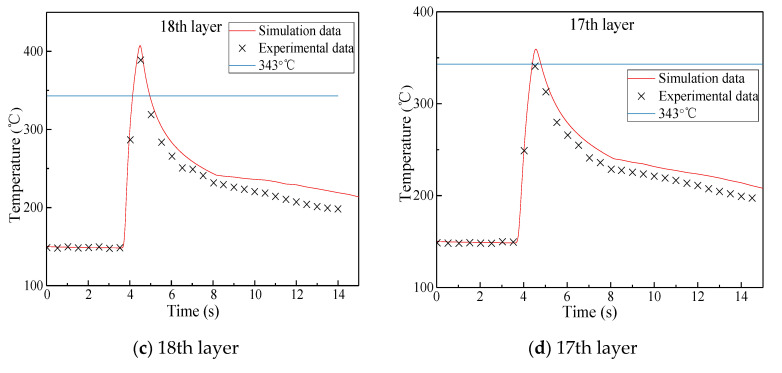
Comparison of experimental results and simulation results at v = 20 mm/s and T = 700 °C.

Figure 14 shows the temperature comparisons of the 20th, 19th, 18th, and 17th layers in the substrate layer of the laminate during heating between the experimental results and the simulation results with a placement speed of v = 20 mm/s and a heating temperature of T = 700 °C. It can be seen that the measured temperature field changes in the substrate of the layup were basically consistent with the results obtained from the simulation analysis, while the difference between maximum temperatures was relatively large. The reasons for this were as follows: firstly, due to the relatively simple settings of some regional boundary conditions of the simulation model, the results obtained from the simulation analysis were higher than those obtained experimentally; secondly, small local bubbles were formed at the thermocouples embedded in each layer during the measurement process, and the air thermal resistance had a great influence on heat transfer in the substrate layer, which resulted in the experimental results being lower than the true value; thirdly, although the mold temperature was maintained around 150 °C in the placement experiment, there existed heat conduction within the mold, the substrate, and the surrounding air, which resulted in the initial temperature of the top layer being lower than that of the bottom layer and the measured initial temperature being lower than the simulated value; lastly, during the placement of the thermoplastic fibers, the temperature field in the substrate layer changed transiently, and the temperature within layers fluctuated rapidly. The temperature change rate of the type K thermocouples had a delay in temperature measurement, while the acquisition time of the measurement system was set to a constant of 0.5 s. These factors may have resulted in the experimental values being lower than the simulation values.

## 5. Conclusions

This paper studied the temperature field changes during the placement of thermoplastic fibers. A two-dimensional heat transfer model during the placement of thermoplastic fibers was established on the basis of heat transfer theory, and the relevant boundary conditions of the heat transfer model were then determined. A finite element model of the temperature field using APDL command flow was established to conduct continuous transient analysis of the substrate layer, investigating the influence of placement process parameters and initial mold temperature on the temperature distribution in the first layer during its placement. Considering the relationship between heating temperature and placement speed, when the initial temperature of the mold was set to 150 °C, the layers could be laid quickly with low speed and high temperature, which was more conducive to interlaminar fusion under the action of the pressing roller. This paper also revealed the mapping relationship between the heating temperature and the temperature field in the substrate, obtaining a reasonable numerical range for each placement process parameter, thereby providing a basis for the selection and setting of the thermoplastic fiber placement parameters. Meanwhile, the influence law of the temperature field in the placement process was obtained, whereby the temperature in any unit of the fiber body increased suddenly at the meshing point, reaching the maximum temperature value before gradually decreasing. Finally, an online temperature detection system in the layers was constructed, and an experiment of real-time temperature acquisition was carried out. The experimental results were basically consistent with the simulation results, with an error of less than 8%, proving the feasibility of the finite element analysis model established in this paper.

## Figures and Tables

**Table 1:** Polyether ether ketone (AS4/PEEK) material properties.

Parameters	Unit	AS4/PEEK
Density *ρ*	kg/m^3^	1560
Specific heat capacity *C*	J/(kg·°C)	1425
Thermal conductivity *K*	W/(m·°C)	*K_x_* = 6 *K_y_* = 0.72
Glass transition temperature *T*_g_	°C	143
Melting temperature *T*_m_	°C	343
Degradation temperature *T*_d_	°C	580
Layer thickness *h*	mm	0.125

**Table 2:** Mold attributes (45# steel).

Parameters	Values
Thermal conductivity *K*	45 W/(m·°C)
Specific heat capacity *C*	470 J/(kg·°C)
Density *ρ*	7800 kg/m^3^

**Table 3:** Parameters in boundary conditions.

Parameter	Value Range	Description
*T*_hg_	500–850 °C	Heating temperature
*v*_x_	2–30 mm/s	Placement speed
*h*_a_	10 W/(m·°C)	Ambient heat transfer coefficient
*h*_hg_	250 W/(m·°C)	Hot gas heat transfer coefficient
*T*_a_	25 °C	Ambient temperature
*T*_r_	80 °C	The roller temperature
*D*	46 mm	The roller diameter
*T*_p_	25 °C	Initial temperature of fiber
*T*_m_	150 °C	Initial temperature of substrate
*T*_s_	150 °C	Initial temperature of layer
*r*_1_	10 mm	Contact length on the fusion right
*r*_2_	3 mm	Contact length on the fusion left
*w*_l_	15 mm	Heated length

## References

[1] Broderick H.C., Paul M.W. (2016). Buckling analysis, design and optimization of variable-stiffness sandwich panels. Int. J. Solids Struct..

[2] Mattia D.F., Laura V., Giuseppe D.A., Kevin P. (2017). Heater power control for multi-material, variable speed Automated fibre placement. Compos. Part A Appl. Sci. Manuf..

[3] Lukaszewicz D., Ward C., Potter K. (2012). The engineering aspects of automated prepreg layup: History present and future. Compos. Part B Eng..

[4] August Z., Ostrander G., Michasiow J. (2014). Recent developments in automated fiber placement of thermoplastic composites. SAMPE J..

[5] Tardif X., Pignon B., Boyard N., Schmelzer J.W., Sobotka V., Delaunay D. (2014). Experimental study of crystallization of PolyEtherEtherKetone (PEEK) over a large temperature range using a nano-calorimeter. Polym. Test.

[6] Mouritz A.P., Leong K.H., Herszberg I. (1997). A review of the effect of stitching on the inplane mechanical properties of fibre-reinforced polymer composites. Compos. Appl. Sci. Manuf..

[7] Morey B. (2008). Automating composites fabrication. Manuf. Eng..

[8] Grouve W.J., Warnet L., Akkerman R., Wijskamp S., Kok J.S. (2010). Weld strength assessment for tape placement. Int. J. Mater. Form..

[9] Qureshi Z., Swait T., Scaife R., Dessouky H.M. (2014). In situ consolidation of thermoplastic prepreg tape using automated tape placement technology: Potential and possibilities. Compos. Part B Eng..

[10] Pedro R., Hamed A., Andrzej T. (2013). A review on the mechanical behaviour of curvilinear fiber composite laminates panels. J. Compos. Mater..

[11] Gearóid C., Daniël P., Vincenzo O., David J., Ronan H., Paul W. (2019). A study of the influence of processing parameters on steering of carbon fibre/PEEK tapes using laser-assisted tape placement. Compos. Part B Eng..

[12] Yang F., Pitchumani R. (2002). Interlaminar contact development during thermoplastic fusion bonding. Polym. Eng. Sci..

[13] Aswani K.B., Gearóid C., Daniël P.B., Ronan M.O. (2019). Properties of a thermoplastic composite skin-stiffener interface in a stiffened structure manufactured by laser-assisted tape placement with in situ consolidation. Compos. Struct..

[14] Dirk H.J., Kevin D.P., Jonathan E. (2013). A concept for the in situ consolidation of thermoset matrix prepreg during automated lay-up. Compos. Part B Eng..

[15] Stokes C.M., Compston P. (2016). Investigation of sub-melt temperature bonding of carbon-fibre/PEEK in an automated laser tape placement process. Compos. Part A Appl. Sci..

[16] Yang F., Pitchumani R. (2003). Nonisothermal healing and interlaminar bond strength evolution during thermoplastic matrix composites processing. Polym. Compos..

[17] Dai S.C., Ye L. (2002). GF/PP tape winding with on-line consolidation. J. Reinf. Plast. Compos..

[18] Sonmez F.O., Hahn H.T. (1997). Analysis of the On-Line consolidation process in thermoplastic composite tape placement. J. Thermoplast. Compos. Mater..

[19] Mantell S.C., Springer G.S. (1992). Manufacturing process models for thermoplastic composites. J. Compos. Mater..

[20] Khan M.A., Mitschang P., Schledjewski R. (2010). Identification of some optimal parameters to achieve higher laminate quality through tape placement process. Adv. Polym. Technol..

[21] Grove S.M. (1988). Thermal modelling of tape laying with continuous carbon fibre-reinforced thermoplastic. Composites.

[22] Kim H.J., Kim S.K., Lee W.I. (2004). Flow and heat transfer analysis during tape layup process of APC-2 prepregs. J. Thermoplast. Compos. Mater..

[23] Schlottermuller M., Lu H., Roth Y. (2003). Thermal residual stress simulation in thermoplastic filament winding process. J. Thermoplast. Compos. Mater..

[24] Rizzolo R.H., Walczyk D.F. (2016). Ultrasonic consolidation of thermoplastic composite prepreg for automated fiber placement. J. Thermoplast. Compos. Mater..

[25] John T., Gillespie J. (2005). Modeling of in situ strength development for the thermoplastic composite tow placement process. J. Compos. Mater..

[26] Ghasemi M.N., Cope R.D., Güceri S. (1991). Thermal analysis of in situ thermoplastic composite tape laying. J. Thermoplast. Compos..

[27] Lionetto F., DellAnna R., Montagna F. (2016). Modeling of continuous ultrasonic impregnation and consolidation of thermoplastic matrix composites. Compos. Part A Appl. Sci..

[28] Dipa R., Anthony J., John L. (2015). Fracture toughness of carbon fiber/polyether ether ketone composites manufactured by autoclave and laser-assisted automated tape placement. J. Appl. Polym. Sci..

[29] Stokes M., Compston P. (2015). The effect of processing temperature and placement rate on the short beam strength of carbon fiber-PEEK manufactured using a laser tape placement process. Compos. Part A Appl. Sci..

[30] Stokes C.M., Compston P. (2015). A combined optical-thermal model for nearinfrared laser heating of thermoplastic composites in an automated tape placement process. Compos. Part A Appl. Sci. Manuf..

[31] Li Y.H., Fu H.Y., Han Z.Y. (2012). Simulation of non-isothermal crystallization kinetics of thermoplastics during fiber placement process. Polym. Polym. Compos..

[32] John T., John W., Gillespie J. (2003). Modeling of heat transfer and void dynamics for the thermoplastic composite tow-placement process. J. Compos. Mater..

[33] Fazil O., Sonmez H., Thomas H. (1997). Modeling of heat transfer and crystallization in thermoplastic composite tape placement process. J. Thermoplast. Compos. Mater..

[34] Noha H., Joseph E., Thompson R. (2005). A heat transfer analysis of the fiber placement composite manufacturing process. J. Reinf. Plast. Comp..

[35] Chinesta F., Leygue A., Bognet B. (2014). First steps towards an advanced simulation of composites manufacturing by automated tape placement. Int. J. Mater. Form..

[36] Toso Y.M., Ermanni P., Poulikakos D. (2004). Thermal phenomena in fiber-reinforced thermoplastic tape winding process: Computational simulations and experimental validations. J. Compos. Mater..

[37] Guan X., Pitchumani R. (2004). Modeling of spherulitic crystallization in thermoplastic towplacement process: Heat transfer analysis. Compos. Sci. Technol..

[38] Tumkor S., Turkmen N., Chassapis C. (2001). Modeling of heat transfer in thermoplastic composite tape lay-up manufacturing. Heat Mass Transf..

